# Small cell carcinoma of the oesophagus: experience of an Indian Tertiary Cancer Centre

**DOI:** 10.3332/ecancer.2022.1393

**Published:** 2022-05-19

**Authors:** Goutam Santosh Panda, Vanita Noronha, Subhash Yadav, Amit Joshi, Vijay Patil, Nandini Menon, Rajiv Kumar, Amit Janu, Abhishek Mahajan, Nilendu Purandare, Jai Prakash Agarwal, George Karimundackal, Kumar Prabhash

**Affiliations:** 1 Department of Medical Oncology, Tata Memorial Centre, Homi Bhabha National Institute, Mumbai 400012, India; 2 Department of Pathology, Tata Memorial Centre, Homi Bhabha National Institute, Mumbai 400012, India; 3 Department of Radiodiagnosis, Tata Memorial Centre, Homi Bhabha National Institute, Mumbai 400012, India; 4 Department of Nuclear Medicine, Tata Memorial Centre, Homi Bhabha National Institute, Mumbai 400012, India; 5 Department of Radiation Oncology, Tata Memorial Centre, Homi Bhabha National Institute, Mumbai 400012, India; 6 Department of Surgical Oncology, Tata Memorial Centre, Homi Bhabha National Institute, Mumbai 400012, India

**Keywords:** small cell carcinoma of the oesophagus, systemic therapy, toxicity, outcome

## Abstract

**Introduction:**

Small cell carcinoma of the oesophagus (SCCE) is a rare and aggressive tumour with no established standard treatment.

**Methods:**

This is a retrospective study of adult patients with histologically proven SCCE registered between February 2011 and March 2020 at Tata Memorial Hospital in Mumbai.

**Results:**

There were 56 patients, with 29 (51.8%) having limited-stage disease (LD) and 27 (48.2%) having extensive-stage disease (ED). The median age was 58 (interquartile range = 51–65) years; 57.1% were men; and 40% were smokers. Among LD-SCCE patients, 23 underwent local therapy, i.e., radiation (19, 65.5%) and surgery (4, 13.8%), and 27 received chemotherapy in neoadjuvant (23, 79.3%), concurrent (18, 62.1%) and adjuvant (4, 13.8%) settings. Totally, 19 ED-SCCE patients (70.4%) received chemotherapy. Prophylactic cranial irradiation (PCI) was delivered to 11 (37.9%) and 7 (25.9%) patients with LD-SCCE and ED-SCCE, respectively. Signiﬁcant grade 3 or more chemotoxicities in patients with LD-SCCE and ED-SCCE included febrile neutropenia in 33.3% and 23.5%, anaemia in 9.5% and 17.6%, and dyselectrolytemia in 14.3% and 11.8%, respectively. The median overall survival (OS) in LD-SCCE and ED-SCCE was 22.9 (95% CI = 1.8–44.1) months and 11.8 (95% CI = 7.3–16.4) months, respectively. Age <60 years (*p* = 0.004) and tumour epicentre in the lower third oesophagus (*p* = 0.002) were independent good prognostic factors for OS in LD-SCCE and ED-SCCE patients, respectively. The incidence of brain metastasis was low, at both presentation (1/27, 3.7%) and relapse (5/56, 8.9%).

**Conclusion:**

Although the survival of LD-SCCE is better than ED-SCCE, it is still under 2 years. Brain metastases are uncommon and the role of PCI is uncertain.

## Introduction

Oesophageal cancer is the sixth most common cancer with an incidence of 5.04% in India as per the WHO GLOBOCAN 2018 [1]. Squamous carcinoma and adenocarcinoma are the common histological types, while small cell carcinoma is rare, having an incidence of 0.1%–2.4% of all oesophageal cancers [2–5]. The first case of primary small cell carcinoma of the oesophagus (SCCE) was described by Mckeown [6] in 1952. SCCE is a highly aggressive malignancy, with approximately half of the patients presenting with metastatic disease [5, 7] and has a poor prognosis [8]. Divergent differentiation of stem cells [9, 10] and development from squamous cell carcinoma [11] are some of hypotheses explaining the origin of SCCE. However, the exact mechanism mediating the development of SCCE remains unclear.

SCCE is thought to behave clinically similarly to small cell lung cancer (SCLC) and the management of SCCE is often extrapolated from small cell lung cancer. Chemotherapy, surgery and radiotherapy have been used with inconsistent outcomes. Randomised studies are not available owing to the rarity of this malignancy. Several retrospective studies have reported differences in survival, depending on the treatment modality [5, 7]. Hence, we retrospectively analysed the available clinical data of SCCE patients registered at our institute and reviewed the literatur in an attempt to define the optimal treatment strategy.

## Methods

### General study details

This is a retrospective analysis of prospectively collected data of all adult patients (age ≥ 18 years) with SCCE who registered at Tata Memorial Hospital in Mumbai, India, between February 2011 and March 2020. Patients with mixed tumours, i.e., SCCE with another histology like squamous carcinoma or adenocarcinoma, were also considered for this study. We excluded patients for whom no clinical details were available. Since the study was retrospective, ethics committee approval and informed consent were not required. No funding support was utilised. The study was conducted according to the ethical principles outlined in the Declaration of Helsinki and the Indian Council of Medical Research Good Clinical Practice Guidelines.

Our primary objective was to determine the overall survival (OS) of patients with SCCE. Our secondary objectives included determining the event-free survival (EFS), evaluating chemotherapy toxicity and prognostic factors affecting survival.

### Study method

**Figure 1.**
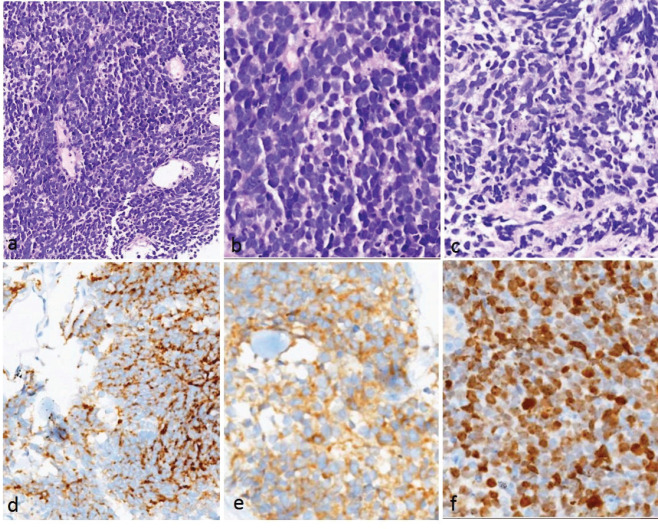
Histopathology and immunohistochemistry of small cell carcinoma of the oesophagus. (a–c): H&E images showing sheets of small round blue cells with hyperchromatic nuclei showing nuclear moulding, inconspicuous nucleoli and scanty/absent cytoplasm. Apoptosis is noted. Immunohistochemistry showed positivity for (d): chromogranin and (e): synaptophysin (e). The proliferation index (Ki-67) was very high (>85%).

The patients included in the study were identified from the rare tumour database maintained in the Department of Medical Oncology and the records maintained in the Department of Pathology. We extracted the data for demographics, clinical and treatment-related details, chemotherapy-related toxicity and survival data. SCCE was diagnosed using the histological criteria laid down by the World Health Organisation (WHO) 2010 classification system [12], and the diagnosis was confirmed by synaptophysin and/or chromogranin A immunostaining (Figure 1).

Workup and management were decided in the multidisciplinary thoracic oncology disease management group tumour board. Tumours were presented as limited-stage disease (LD) or extensive-stage disease (ED) as per the Veteran’s Administration Lung Group staging system for SCLC [13]. The Union for International Cancer Control 1987 standard was used to describe tumour location in the oesophagus. All preliminary evaluation details, including the clinical history, physical examination, endoscopic upper gastrointestinal imaging findings, the epicentre of the tumour in the oesophagus (cervical, upper-third, middle-third, lower-third and abdominal) and imaging results, including computed tomography (CT), magnetic resonance imaging (MRI) brain and/or fluorodeoxyglucose positron emission tomography-computed tomography (FDG PET-CT) findings, complete blood count, renal function tests and liver function tests, were documented and entered into an excel sheet. The treatment strategies, use of prophylactic cranial irradiation (PCI), response to treatment as per Response Evaluation Criteria in Solid Tumours 1.1 (RECIST 1.1) and grade 3 or more toxic events as per CTCAE v 4 [14] were recorded.

### Statistical analysis

Analysis was carried out using the Statistical Package for the Social Sciences (IBM Corp. Released 2016. IBM SPSS Statistics for Windows, Version 24.0. Armonk, NY, IBM Corp.) and R version 3.6.3 cmprsk, survminer, prodlim and risk regression packages from the Comprehensive R Archive Network (R Core Team, 2019). Kaplan–Meier method was used for estimating survival [15] and log-rank test was employed for comparison. Factors significant on univariate analysis were subsequently subjected to multivariate analysis using the Cox regression method [16, 17].

EFS was calculated as the time from registration to the first event, where events included progression, recurrence, second malignancy or death. We have analysed and reported the EFS and OS. Censoring was carried out for OS analysis in case the patients were lost to follow-up longer than 6 months. Censoring for EFS analysis was carried out for patients who had not experienced an event and were lost to follow-up longer than 6 months. OS was calculated from date of registration until death from any cause or last documented follow-up with appropriate censoring. The data cut-off date was 10 December 2020.

## Results

### Patients and tumour characteristics

Of 8342 patients with oesophageal malignancies, 59 (0.7%) had SCCE. No clinical details could be retrieved for three SCCE patients and they were therefore excluded. We included 56 patients in the study (Table 1). Dysphagia (54 cases, 96.4%) was the commonest presenting symptom with a median duration of 2 months before presentation. Two patients (3.6%) had a family history of malignancy.

The tumours commonly arose in the middle and lower third of the oesophagus. FDG PET-CT was performed in 40 (71.4%) patients; contrast-enhanced CT instead of PET-CT was performed in 14 (25%) as there was obvious metastasis in CT scan; and 2 (3.6%) patients defaulted before completion of staging work up. However, these two defaulters had CT thorax showing metastatic disease. Brain imaging at baseline was available as MRI brain in 14 (25%) cases and FDG PET-CT in 30 (53.6%) cases, while in the remaining 12 patients (21.4%) no cross-sectional brain imaging was available. Of the total 56 patients analysed, 29 (51.8%) had LD-SCCE and 27 (48.2%) had ED-SCCE.

### Pathology findings

The tumours showed uniform histology in all the cases, characterised by crushed hyperchromatic cells with scant to absent cytoplasm, prominent nuclear moulding and absent to inconspicuous nucleoli. The immunohistochemistry panel utilised for diagnosis included synaptophysin, chromogranin and CD56. All patients had at least two of the above three positive to confirm the neuroendocrine nature of the tumour. The proliferation (Ki-67) index was high (>70%) in all these tumours (Figure 1).

### Treatment

**Figure 2.**
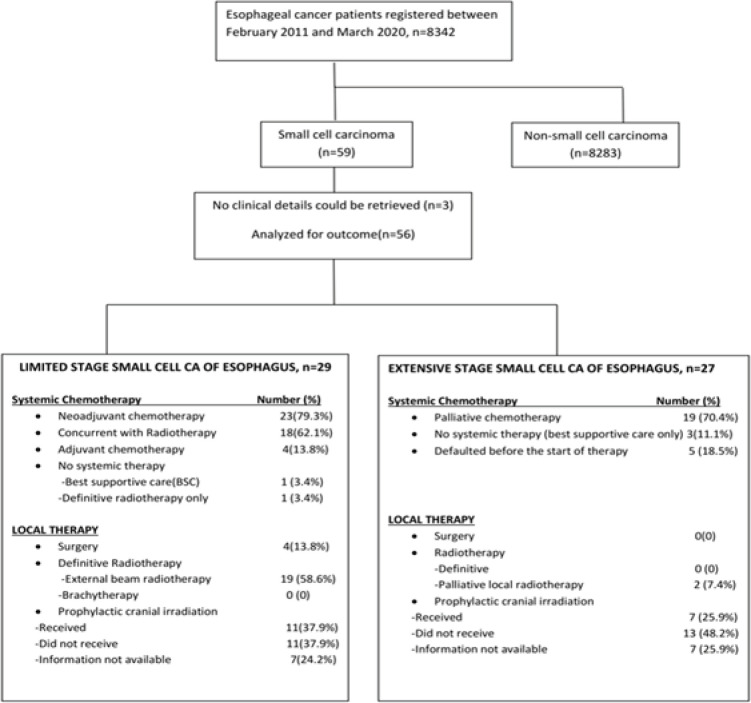
Treatment details of small cell carcinoma of the oesophagus.

**LD-SCCE**: Of 29 patients, 28 (96.6%) were considered for curative intent therapy, while 1 patient (3.4%) was declared best supportive care upfront because of poor Eastern Cooperative Oncology Group (ECOG) performance status (PS). Of 28 patients considered for tumour-directed therapy, local therapy was delivered in 23 (radiotherapy in 19 and surgery in 4). The remaining five patients who were planned for but did not undergo any local therapy included three patients who defaulted post-neoadjuvant chemotherapy (NACT) and two patients who died from chemotherapy-related toxicities on NACT. NACT and adjuvant chemotherapy were administered in 23 (79.3%) and 4 (13.8%) cases, respectively. Concurrent chemoradiation (CRT) was planned for all patients undergoing radiation as local therapy, except one patient, who was deemed unfit for chemotherapy (Figure 2). Eleven (37.9%) patients received PCI.

**ED-SCCE:** Although 24 of the 27 patients (88.9%) with ED-SCCE were advised chemotherapy, 19 (70.4%) received palliative chemotherapy and 7 (25.9%) patients received PCI.

### Chemotherapy

The once-in-3-weeks etoposide and platinum (cisplatin/carboplatin) (EP) doublet regimen was used in 44 out of 46 patients (95.6%) who received chemotherapy. Carboplatin was administered to 28 (60.9%) (13 (28.3%) with LD-SCCE and 15 (32.6%) with ED-SCCE) out of 46 patients receiving chemotherapy. The reasons for using carboplatin instead of cisplatin included low glomerular filtration rate (GFR) (9 in LD-SCCE and 5 in ED-SCCE), advanced age and the clinician’s judgment regarding chemotherapy tolerance (3 in LD-SCCE and 8 in ED-SCCE) and comorbidity (1 in LD-SCCE and 2 in ED-SCCE). One patient received once-a-week paclitaxel and carboplatin as concurrent CRT and one received the same regimen in the palliative setting. The median number of EP cycles in patients with LD-SCCE and ED-SCCE were 4 and 6, respectively.

### Response rate

Radiological responses to treatment were available for 15 of 19 (78.9%) patients with LD-SCCE undergoing non-surgical modality of therapy and 15 of 19 (78.9%) patients with ED-SCCE who received chemotherapy. Responses in patients with LD-SCCE and ED-SCCE included complete response (CR) in 9/15 (60%) and 2/15 (13.3%), partial response in 5/15 (33.3%) and 8/15 (53.3%), stable disease in 0/15 and 3/15 (20%) and progressive disease in 1/15 (6.67%) and 2/15 (13.3%), respectively.

### Surgery

Five LD-SCCE patients were planned for surgery. Of these, four underwent oesophagectomy with three-ﬁeld lymph node dissection (cervical, mediastinal and perigastric lymph nodes), while one patient defaulted post-NACT. All four patients had R0 resection and had received NACT with EP – three cycles in two cases, four cycles in one case and six cycles in one case. Histopathology revealed scanty residual tumour cells in one case and one focus of *in situ* carcinoma in one case, while there was evidence of residual invasive carcinoma in the remaining two cases. None of these patients received adjuvant chemotherapy.

### Radiotherapy

The median dose of radiotherapy delivered as external beam radiotherapy (EBRT) in LD-SCCE was 63 Gy/35# to locoregional site. All received conventional fraction EBRT with 45 Gy/25# in 1, 50.4 Gy/28# in 2, 55.8 Gy/31# in 1, 59.4 Gy/33# in 1, 60 Gy/30# in 3 and 63 Gy/35# in the remaining 11 patients. Two patients with ED-SCCE received palliative RT to the local site. The PCI dose regimens included 24.75 Gy/11# in six patients, 25 Gy/10# and 24 Gy/8# in two each with a median dose of 24.75 Gy/11# in both LD-SCCE and ED-SCCE.

### Chemotherapy-related toxicities

**LD-SCCE**: The toxicity data were available for 21 patients. Signiﬁcant grade 3 or more haematological toxicities were febrile neutropenia (FN) in 7/21 (33.3%), thrombocytopenia in 2/21 (9.5%) and anaemia in 2/21 (9.5%), while the non-haematological toxicities included chemotherapy-induced nausea and vomiting (CINV) and fatigue in 2/21 each (9.5%), gastrointestinal (GI) toxicity in 4/21 (19.0%), dyselectrolytemia in 3/21 (14.3%) and hepatorenal syndrome in 1/21 (4.8%). Four patients (4/21, 19%) required dose modification or omission during chemotherapy. Deaths attributable to chemotherapy occurred in 2/21 patients (9.5%), one due to febrile neutropenia and another due to hepatorenal syndrome. Overall, nine patients (42.9%) experienced grade 3 or more chemotoxicity.

**ED-SCCE:** Toxicity data were available for 17 out of 27 patients with ED-SCCE. Notable haematological grade 3 or more toxicities were FN in 4/17 (23.5%), thrombocytopenia in 1/17 (5.9%) and anaemia in 3/17 (17.6%), while the non-haematological toxicities included CINV in 1/17 (5.9%), fatigue in 3/17 (17.6%), GI toxicity in 1/17 (5.9%), dyselectrolytemia in 2/17 (11.8%) and renal toxicity in 1/17 (5.9%). Six (6/17, 35.3%) patients required dose modification or omission during chemotherapy. No patient with ED-SCCE died of chemotherapy toxicity. Overall, 10 patients (58.8%) experienced grade 3 or more chemotherapy-related toxicity.

### Survival

**Figure 3.**
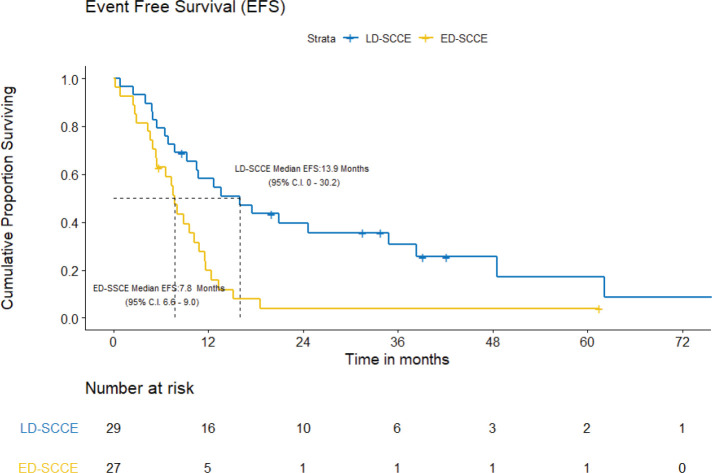
EFS of limited-stage disease LD-SCCE and extensive-stage disease ED-SCCE.

**Figure 4.**
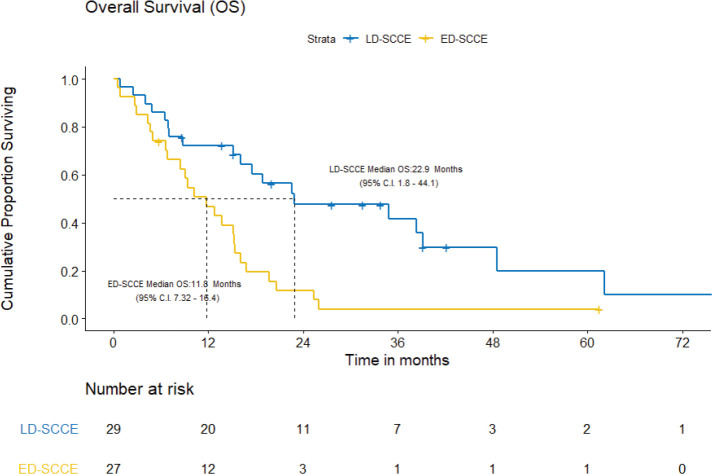
OS of limited-stage disease LD-SCCE and extensive-stage disease ED-SCCE.

With a median follow-up of 42.2 (IQR = 20–61.5) months, the median EFS and OS for the entire cohort were 10.2 (95% CI = 7.5–12.9) months and 15.4 (95% CI = 12.7–18.2) months, respectively. Figures 3 and 4 show the EFS and OS of the two cohorts, i.e., LD-SCCE and ED-SCCE, respectively. 16 (28.6%) patients (LD-SCCE: 9 patients and ED-SCCE: 7 patients) had follow-up less than 2 years.

**LD-SCCE**: The median follow-up in surviving patients was 39.2 (IQR = 27.6–105.6) months.

A) EFS and patterns of failure: At the time of analysis, 22 (75.9%) patients experienced events for EFS (recurrence/progression/deaths: 17/2/3). Of 19 recurrences/progressions, 2 had both locoregional and distant failures and 17 had distant-only failures. Only 2 patients failed in the brain. The median EFS was 15.9 (95% CI = 0–30.2) months. The estimated 2-year EFS was 39.6%.

B) OS: There were 19 deaths, including the 2/29 (6.9%) chemotherapy-toxic deaths and 1/29 (3.5%) death due to sepsis post-surgery. The median OS was 22.9 (95% CI = 1.8–44.1) months and the 2-year OS estimate was 47.7%.

**ED-SCCE**: The median follow-up in surviving patients was 24.4 (IQR = 8.5–61.5) months.

A) EFS and patterns of failure: At the time of analysis, 25 (92.6%) patients had experienced events for EFS (recurrence: 22; progression: 2; and death due to non-cancerous cause: 1). The median EFS was 7.8 (95% CI = 6.6–9.0) months. The projected 2-year EFS was 3.9%.

B) OS: The median OS was 11.8 (95% CI = 7.3–16.4) months. The estimated OS at 2 years was 11.7%.

### Relapse in brain

Of all the patients with LD-SCCE, 18 (62.1%) patients completed the planned therapy without disease progression. Similarly, PCI was considered for 10 (37%) patients with ED-SCCE who did not have brain metastasis and attained CR or PR at the completion of first line of chemotherapy. Of these 28 patients, 18 (64.3%) received PCI (LD-SCCE: 11 and ED-SCCE: 7). A total of five patients relapsed in the brain (LD-SCCE: 2 and ED-SCCE: 3) (Figure 4).

### Prognostic factors

**Entire cohort**: In univariate analysis, female sex, history of substance abuse, palliative intent treatment and raised LDH were predictors for poor EFS. History of substance abuse was the only independent prognostic factor for EFS, while no factor was independently prognosticated for OS (Table 2).

## Discussion

In our SCCE cohort of 56 patients, the median OS was 22.9 (95% CI = 1.8–44.1) months for LD-SCCE and 11.8 (95% CI = 7.3–16.4) months for ED-SCCE. As per Ding *et al* [18], LD-SCCE patients who underwent surgery and post-operative chemotherapy achieved a median OS of 26 months. Moreover, the use of chemotherapy in combined modality treatment doubled the median OS from 11 months to 22 months (HR = 2.30; *p* = 0.001). As per the published literature, the median OS of patients with LD-SCCE treated with a multimodality approach ranges from 14 months to as high as 39.7 months [18–22]. In our study, only one patient with LD-SCCE was offered single modality radical therapy (radiotherapy) as he was unfit for systemic therapy. Hence, we were unable to estimate the incremental benefit of systemic therapy in our patients with LD-SCCE. In a systematic analysis of 19 SCCE case series, the median OS in 88 patients with ED-SCCE was 9 months [23]. The OS of ED-SCCE was similar to other studies [9, 20, 23, 24]. Thus, the outcomes of our patients with LD-SCCE and ED-SCCE were similar to those in the published literature. Because both pulmonary and extrapulmonary small cell cancers are systemic diseases, localised treatment alone offers only limited survival; therefore, multimodality therapy is preferred even at an early stage. This is reflected in our patients’ increased distant relapse (17 of 19 recurrences/progressions had distant-only failure), indicating it to be a systemic disease and emphasising the need for better systemic therapy for these patients. Both surgery and chemo-radiation result in good local control. In addition, in the ED-SCCE, radiotherapy may aid in the palliation of dysphagia.

In our study, approximately half the patients (30 out of 66) had a history of either smoking or alcohol consumption. History of smoking was reported in 50% of small cell oesophagus patients in a research conducted in North-east India [25]. While there are no well-defined risk factors for SCCE, they appear to be similar to those for squamous cell oesophageal cancer (history of alcohol consumption and smoking) [26, 27]. Smoking has been identified as a significant risk factor in patients with SCLC, with a history of smoking being elicited in more than 90% of the cases [28, 29]. In our cohort of patients with SCCE, smoking appeared to be less important as an aetiological factor. As per the Global Adult Tobacco Survey, smoking is less common in India, with 19% of men and 2% of women being smokers [30]. In our earlier studies, we had noted that 10%–15% of our patients with SCLC and 55% of the patients overall with lung cancer were non-smokers [31–33].

The optimal locoregional treatment for LD-SCCE is controversial. There are several reports and meta-analyses that recommend surgery as the local therapy of choice [3, 34–36]. Regarding radiotherapy, several studies have reported that CRT can also achieve long-term survival in LD-SCCE patients [22, 37, 38]. A retrospective study that directly compared surgery and radiotherapy as local treatments found no difference in survival or locoregional recurrence between patients treated with the two modalities [20]. In our study, radiotherapy was the local treatment in majority (65.5%) of the LD-SCCE patients (only 4 (13.8%) patients underwent resection). Given the non-randomised nature and small sample size of this study, comparing the outcomes of patients treated with radiotherapy to surgery is inappropriate. However, the findings of our study indicate that CRT may be regarded as an alternative radical treatment strategy in LD-SCCE, with the benefit of preventing surgical complications and delaying the start of post-operative chemotherapy [20, 22, 39].

The most common chemotherapy-related toxicity was febrile neutropenia seen in 30% of the patients in our study and the overall rate of grade 3 or more toxicity was 50%. In a study by Chen *et al* [22], the overall rate of grade 3–4 toxicities was 37.5%. There is a paucity of chemotherapy toxicity data as most studies are retrospective in nature and focused on outcomes rather than adverse events [21–24]. We observed that older age was associated with inferior OS in our LD-SCCE patients. Analysis of the SEER database also found that older age was associated with a worse OS [5, 40]. Other researchers have also made similar observations, albeit the association was not statistically significant [22, 41]. Additionally, older age has been reported as a poor prognostic factor in SCLC [42]. Patients with a history of substance abuse had worse EFS in our cohort. However, a history of smoking had no impact on survival in other studies [22, 43]. Among our patients with ED-SCCE, those with a primary site in the lower-third oesophagus had better outcomes than others. An earlier study described the origin in the middle-third oesophagus as a predictor for better OS in univariate analysis [43], while others did not find any significant correlation of primary tumour location and outcome [44, 45]. Small sample sizes of the various studies and different tumour biologies may be the reasons for the apparent differences in prognostic factors.

PCI is practiced in SCCE as an extrapolation of the data from SCLC, where the incidence of brain metastasis is as high as 50–80% at 2 years [46]. According to previous studies, the rate of brain metastasis in SCCE is 5%–6%, which is much lower than the rate in SCLC [37]. In our study, only 1 (3.7%) patient with ED-SCCE had brain metastasis at presentation. We also observed the brain relapse rate was much lower than what has been historically reported in patients with SCLC. We observed that the proportions of patients developing brain metastasis in both LD-SCCE and ED-SCCE were similar, irrespective of PCI. Hence, PCI may be avoided cautiously in patients with SCCE with close clinicoradiological follow-up. This hypothesis is supported by other studies as well [9, 21, 47].

The main limitations of our study are the small sample size, retrospective nature and single-institution study. Since none of our patients received immunotherapy, we are unable to determine the efficacy of immune checkpoint inhibitors in SCCE. Some of the records, such as those relating to past history, environmental exposures and quality of life, relief of dysphagia with systemic therapy were limited. The availability of detailed systemic therapy toxicity data in the majority of patients is strength of our study. However, toxicity of radiation and surgery has not been included. The rarity of this malignancy makes the conduct of a randomised trial difficult. In such cases, retrospective studies and clinicians’ experiences have become important means to guide management.

## Conclusion

Although the survival of LD-SCCE is better than ED-SCCE, it is still under 2 years with ample room for improvement. Brain metastasis is not common and the role of PCI is uncertain. National and international collaborations are needed to conduct prospective studies to bridge the gap in the knowledge of treatment and prognostic factors in this rare tumour.

## Funding

No research support was received for this study.

## Conflicts of interest

The authors declare that they have no known competing financial interests or personal relationships that could have appeared to influence the work reported in this paper.

## Figures and Tables

**Figure 5.**
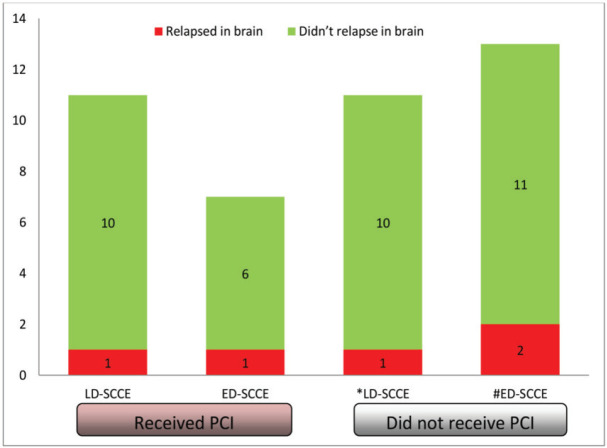
Categorisation of patients relapsing in brain. Abbreviations: LD-SCCE = Limited-stage disease small cell carcinoma of the oesophagus; ED-SCCE = Extensive-stage disease small cell carcinoma of the oesophagus; and PCI: prophylactic cranial irradiation. *PCI status of the remaining seven patients not known. #PCI status of the remaining seven patients not known.

**Table 1.** Baseline characteristics.

Variable	LD-SCCE, number (%), *n* = 29	ED-SCCE, number (%), *n* = 27
**Patient characteristics**		
1. Age (in years) Median Interquartile range (IQR)	58 50.5–66.5	57 51–64
2. Sex Male Female	18 (62.1%) 11 (37.9%)	15 (55.6%) 12 (44.4%)
3. Comorbidity Yes No	14 (48.3%) 15 (51.7%)	10 (37.1%) 17 (62.9%)
4. ECOG PS 0–1 ≥2	28 (96.5%) 1 (3.5%)	21 (77.8%) 6 (22.2%)
5. Addiction^a^ Tobacco chewer Smoker Alcohol None	14 (48.3%) 11 (37.9%) 2 (6.9%) 4 (13.8%)	11 (40.7%) 12 (44.4%) 5 (18.5%) 4 (14.8%)
**Tumour characteristics**		
6. Location Cervical + upper Middle Lower + GEJ	4 (13.8%) 18 (62.1%) 7 (24.1%)	3 (11.1%) 13 (48.1%) 11 (40.7%)
7. Histology Pure small cell carcinoma Mixed histology	26 (89.7%) 3 (10.3%)	25 (92.6%) 2 (7.4%)
9. Median length in cm	6.2	7.4
10. Skip lesion Yes No	1 (3.4%) 28 (96.6%)	2 (7.4%) 25 (92.6%)
11. Regional lymph node involvement Yes No	26 (89.7%) 3 (10.3%)	26 (96.3%) 1 (3.7%)
12. Metastatic sites Liver Non-regional lymph node Lungs Bone Bone marrow Brain	NA NA NA NA NA NA	17 (62.9%) 13 (48.1%) 7 (25.9%) 6 (22.2%) 5 (18.5%) 1 (3.7%)
13.Number of metastatic sites 1 2 ≥3	NA NA NA	13 (48.1%) 7 (25.9%) 7 (25.9%)
**Laboratory parameters** **Median (IQR) Median (IQR)**		
14. Haemoglobin in gm/dl	12.3 (11.3–14.3)	12.6 (11.6–14.1)
15. Albumin in gm/dl	4.1 (3.8–4.3)	3.9 (3.6–4.2)
16. Serum alkaline phosphatasein U/L	98 (81–113)	112 (87–148.5)
17. LDH in U/L	170 (152–196.5)	328 (203–689)

**Abbreviations**: LD-SCCE: Limited-stage disease small cell carcinoma of the oesophagus; ED-SCCE: Extensive-stage disease small cell carcinoma of the oesophagus; ECOG PS: Eastern Cooperative Oncology Group performance status; GEJ: Gastroesophageal junction; NA: Not applicable; LDH: Lactate dehydrogenase

a Sum is more than the total number of patients as few patients had a history of more than one type of substance abuse

**Table 2.** Significant factors of univariate and multivariate EFS and OS analyses.

		Whole cohort (n = 56)	LD-SCCE (n = 29)	ED-SCCE (n = 27)
		**[HR=, *p*=]**	**[HR, *p*=]**	**[HR, *p*=]**
**EFS, univariate analysis**				
Age at diagnosis (in years)	≥60	–		–
	<60	–	HR = 0.2, *p* = 0.02	–
History of substance abuse	Yes			–
	No	HR = 0.3, *p* = 0.004	HR = 0.65, *p* = 0.03	–
Gender	Female		–	–
	Male	HR = 0.5, *p* = 0.006	–	–
LDH	Raised		–	–
	Not raised	HR = 0.3, *p* = 0.004	–	–
Intent of treatment	Palliative		–	–
	Curative	HR = 0.3, *p* < 0.001	–	–
**EFS, multivariate analysis**				
History of substance abuse	Yes		–	–
	No	HR = 0.12, *p* = 0.001	–	–
**OS, univariate analysis**				
Age at diagnosis (in years)	≥60	–		
	<60	–	HR = 0.2, *p* = 0.004	–
ECOG PS	>1		–	–
	0-1	HR = 0.44, *p* = 0.04	–	–
Intent of treatment	Palliative		–	–
	Curative	HR = 0.3, *p* = 0.00	–	–
LDH	Raised		–	–
	Not raised	HR = 0.32, *p* = 0.007	–	–
Site of primary	Cervical, upper 1/3^rd^		–	
	middle-third		–	Worst prognosis
	lower-third, abdominal		–	HR = 0.3, *p* = 0.002 (Best prognosis)
**OS, multivariate analysis**				
No significant prognostic factor				

**Abbreviations**: LD-SCCE: Limited-stage disease-small cell carcinoma of the oesophagusl; ED-SCCE: Extensive-stage disease-small cell carcinoma of the oesophagus; HR: Hazard ratio; EFS: Event-free survival; OS: Overall survival; ECOG PS: Eastern Cooperative Oncology Group performance status; LDH: Lactate dehydrogenase
